# [Cu_2_(HF_2_)(H_2_O)_8_][AlF_6_]·2H_2_O

**DOI:** 10.1107/S1600536809009702

**Published:** 2009-03-19

**Authors:** Matthias Weil

**Affiliations:** a Institute for Chemical Technologies and Analytics, Division of Structural Chemistry, Vienna University of Technology, Getreidemarkt 9/164-SC, A-1060 Vienna, Austria

## Abstract

The title compound, octaaqua­(hydrogendifluorido)­di­cop­per(II) hexa­fluoridoaluminate dihydrate, was obtained under hydro­thermal conditions. The structure is isotypic with that of the analogous Fe^III^ compound, [Cu_2_(HF_2_)(H_2_O)_8_][FeF_6_]·2H_2_O. The coordination sphere of the Cu^II^ atom is formed by one F and three water O atoms at short distances < 2 Å and is augmented by two additional water O atoms at significantly longer distances, leading to a considerably distorted octa­hedral environment. By edge-sharing, these octa­hedra form dimeric [Cu_2_(HF_2_)(H_2_O)_8_]^3+^ units that are bonded to [AlF_6_]^3−^ anions (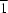 symmetry) and to crystal lattice water mol­ecules *via* hydrogen bonds. Besides F—H⋯F inter­actions between the dimeric cationic units, O—H⋯F and O—H⋯O hydrogen bonds (both in part bifurcated) are observed.

## Related literature

For the structure of the isotypic Fe^III^ analogue, see: Le Bail & Mercier (2009 ▶). For a natural compound in the Cu/Al/F/O/H system, Cu_4_Al_3_(OH)_14_F_3_(H_2_O)_2_ (mineral name khaidarkanite), see: Rastsvetaeva *et al.* (1997 ▶).

## Experimental

### 

#### Crystal data

[Cu_2_(HF_2_)(H_2_O)_8_][AlF_6_]·2H_2_O
*M* *_r_* = 487.23
Triclinic, 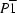

*a* = 6.6119 (3) Å
*b* = 7.3410 (3) Å
*c* = 8.3174 (3) Å
α = 107.336 (1)°
β = 106.715 (1)°
γ = 94.454 (1)°
*V* = 363.15 (3) Å^3^
*Z* = 1
Mo *K*α radiation
μ = 3.12 mm^−1^
*T* = 293 K
0.18 × 0.14 × 0.06 mm

#### Data collection

Bruker SMART CCD area-detector diffractometer
Absorption correction: multi-scan (*SADABS*; Bruker, 2000 ▶) *T*_min_ = 0.52, *T*_max_ = 0.80
3899 measured reflections
2013 independent reflections
1952 reflections with *I* > 2σ(*I*)
*R*_int_ = 0.019

#### Refinement

*R*[*F* ^2^ > 2σ(*F* ^2^)] = 0.026
*wR*(*F* ^2^) = 0.072
*S* = 1.08
2013 reflections
130 parameters
10 restraints
H atoms treated by a mixture of independent and constrained refinement
Δρ_max_ = 0.44 e Å^−3^
Δρ_min_ = −0.54 e Å^−3^

### 

Data collection: *SMART* (Bruker, 2000 ▶); cell refinement: *SAINT* (Bruker, 2000 ▶); data reduction: *SAINT*; program(s) used to solve structure: *SHELXS97* (Sheldrick, 2008 ▶); program(s) used to refine structure: *SHELXL97* (Sheldrick, 2008 ▶); molecular graphics: *ATOMS for Windows* (Dowty, 2006 ▶); software used to prepare material for publication: *SHELXL97*.

## Supplementary Material

Crystal structure: contains datablocks I, global. DOI: 10.1107/S1600536809009702/br2101sup1.cif

Structure factors: contains datablocks I. DOI: 10.1107/S1600536809009702/br2101Isup2.hkl

Additional supplementary materials:  crystallographic information; 3D view; checkCIF report

## Figures and Tables

**Table 1:** Selected bond lengths (Å)

Cu—F4	1.9049 (12)
Cu—O1	1.9441 (14)
Cu—O2	1.9522 (14)
Cu—O3	1.9739 (13)
Cu—O4	2.3463 (15)
Cu—O3^i^	2.7139 (16)
Al—F1	1.8001 (10)
Al—F2	1.8091 (11)
Al—F3	1.8209 (11)

Symmetry code: (i) 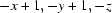.

**Table 2:** Hydrogen-bond geometry (Å, °)

*D*—H⋯*A*	*D*—H	H⋯*A*	*D*⋯*A*	*D*—H⋯*A*
O1—H11⋯F1^ii^	0.87 (3)	1.74 (3)	2.6042 (17)	171 (4)
O1—H12⋯F2	0.83 (3)	1.86 (3)	2.6899 (18)	176 (4)
O2—H21⋯F1^i^	0.81 (3)	1.79 (3)	2.6020 (18)	172 (4)
O2—H22⋯O5^iii^	0.83 (3)	1.86 (3)	2.684 (2)	178 (4)
O3—H31⋯F3^i^	0.77 (3)	1.82 (3)	2.5877 (18)	178 (4)
O3—H32⋯F3^iv^	0.79 (3)	1.89 (3)	2.6700 (17)	174 (4)
O4—H41⋯F2^v^	0.85 (3)	1.95 (3)	2.7840 (19)	169 (4)
O4—H42⋯O4^vi^	0.67 (3)	2.41 (4)	2.758 (3)	115 (4)
O4—H42⋯O5^iv^	0.67 (3)	2.41 (4)	2.784 (2)	117 (4)
O5—H51⋯F2^vii^	0.75 (3)	2.14 (3)	2.8538 (19)	159 (4)
O5—H51⋯F3^viii^	0.75 (3)	2.50 (3)	3.072 (2)	135 (4)
O5—H52⋯F4	1.04 (3)	1.67 (3)	2.6620 (19)	158 (3)
F4—H6⋯F4^v^	0.9031 (13)	1.6945 (13)	2.596 (3)	175.27 (5)

Symmetry codes: (i) 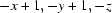; (ii) 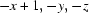; (iii) 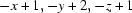; (iv) 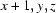; (v) 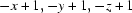; (vi) 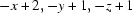; (vii) 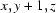; (viii) 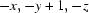.

**Fig. 1.**
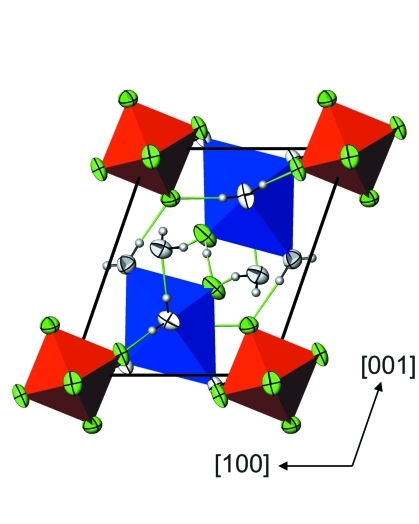
The crystal structure of [Cu2(HF2)(H2O)8][AlF6](H2O)2 in polyhedral representation projected along [010]. Colour key: O atoms white, F atoms green, H atoms are grey, [CuO5F] octahedra are blue, [AlF6] octahedra are red. Displacement ellipsoids are given at the 74% probability level; H atoms are displayed as spheres of arbitrary radius. Hydrogen bonds are indicated with green lines. Note that for clarity only one of the disordered H atoms bonded to the F atom is shown.

## supplementary crystallographic information

### Comment

The crystal structure (Fig. 1) of the title compound, [Cu_2_(HF_2_)(H_2_O)_8_][AlF_6_].2H_2_O), is isotypic with [Cu_2_(HF_2_)(H_2_O)_8_][FeF_6_].2H_2_O (Le Bail & Mercier, 2009). Except the Al—F distances (*¯d* = 1.810 Å *versus* 1.930 Å for the average Fe—F distance), all other interatomic distances, angles and the hydrogen bond geometry are very similar for the two structures. A detailed description of this structure has been given Le Bail & Mercier (2009).

There is one additional compound described in the Cu/Al/F/O/H system, *viz* the mineral khaidarkanite with formula Cu_4_Al_3_(OH)_14_F_3_(H_2_O)_2_ (Rastsvetaeva *et al.*, 1997). The latter differs from the title compound as its structure contains distorted [Cu(OH)_5_(H_2_O)] octahedra, and [Al(OH)_6_] and [AlF_4_(H_2_O)_2_] octahedra as building units.

### Experimental

AlF_3_ and CuSO_4_^.^5H_2_O (both Merck, p.a.) were reacted hydrothermally in a 2 *M* HF solution at 393 K for 4 d. Blue crystals of the title compound with mostly platy habit and up to 0.3 mm in length were obtained.

### Refinement

The structure was solved using direct methods. For better comparison with the isotypic Fe^III^ analogue (Le Bail & Mercier, 2009), the atomic coordinates of the latter were used for the final refinement cycles. All H atoms were located from difference Fourier maps. The water H atoms were restrained to have O—H distances of 0.85 Å. Their *U*_iso_ values were refined with one common parameter. The position of the H atom of the disordered HF group (set with a site occupation factor of 1/2) was fixed during refinement, but its *U*_iso_ value was refined independently.

### Crystal data

**Table:**

[Cu_2_(HF_2_)(H_2_O)_8_][AlF_6_]·2H_2_O	*Z* = 1
*M**_r_* = 487.23	*F*(000) = 244
Triclinic, *P*1	*D*_x_ = 2.228 Mg m^−^^3^
Hall symbol: -P 1	Mo *K*α radiation, λ = 0.71073 Å
*a* = 6.6119 (3) Å	Cell parameters from 3443 reflections
*b* = 7.3410 (3) Å	θ = 2.7–30.0°
*c* = 8.3174 (3) Å	µ = 3.12 mm^−^^1^
α = 107.336 (1)°	*T* = 293 K
β = 106.715 (1)°	Plate, blue
γ = 94.454 (1)°	0.18 × 0.14 × 0.06 mm
*V* = 363.15 (3) Å^3^	

### Data collection

**Table:**

Bruker SMART CCD area-detector diffractometer	2013 independent reflections
Radiation source: fine-focus sealed tube	1952 reflections with *I* > 2σ(*I*)
graphite	*R*_int_ = 0.019
ω scans	θ_max_ = 30.0°, θ_min_ = 2.7°
Absorption correction: multi-scan (*SADABS*; Bruker, 2000)	*h* = −9→9
*T*_min_ = 0.52, *T*_max_ = 0.80	*k* = −10→10
3899 measured reflections	*l* = −11→11

### Refinement

**Table:**

Refinement on *F*^2^	Secondary atom site location: difference Fourier map
Least-squares matrix: full	Hydrogen site location: inferred from neighbouring sites
*R*[*F*^2^ > 2σ(*F*^2^)] = 0.026	H atoms treated by a mixture of independent and constrained refinement
*wR*(*F*^2^) = 0.072	*w* = 1/[σ^2^(*F*_o_^2^) + (0.0482*P*)^2^ + 0.1517*P*] where *P* = (*F*_o_^2^ + 2*F*_c_^2^)/3
*S* = 1.08	(Δ/σ)_max_ = 0.001
2013 reflections	Δρ_max_ = 0.44 e Å^−^^3^
130 parameters	Δρ_min_ = −0.54 e Å^−^^3^
10 restraints	Extinction correction: *SHELXL97* (Sheldrick, 2008), Fc^*^=kFc[1+0.001xFc^2^λ^3^/sin(2θ)]^-1/4^
Primary atom site location: structure-invariant direct methods	Extinction coefficient: 0.059 (5)

### Special details

**Table:**

Geometry. All e.s.d.'s (except the e.s.d. in the dihedral angle between two l.s. planes) are estimated using the full covariance matrix. The cell e.s.d.'s are taken into account individually in the estimation of e.s.d.'s in distances, angles and torsion angles; correlations between e.s.d.'s in cell parameters are only used when they are defined by crystal symmetry. An approximate (isotropic) treatment of cell e.s.d.'s is used for estimating e.s.d.'s involving l.s. planes.
Refinement. Refinement of *F*^2^ against ALL reflections. The weighted *R*-factor *wR* and goodness of fit *S* are based on *F*^2^, conventional *R*-factors *R* are based on *F*, with *F* set to zero for negative *F*^2^. The threshold expression of *F*^2^ > σ(*F*^2^) is used only for calculating *R*-factors(gt) *etc*. and is not relevant to the choice of reflections for refinement. *R*-factors based on *F*^2^ are statistically about twice as large as those based on *F*, and *R*- factors based on ALL data will be even larger.

### Fractional atomic coordinates and isotropic or equivalent isotropic displacement parameters (Å^2^)

**Table:**

	*x*	*y*	*z*	*U*_iso_*/*U*_eq_	Occ. (<1)
Cu	0.60564 (3)	0.55637 (3)	0.23399 (2)	0.01836 (10)	
Al	0.0000	0.0000	0.0000	0.01611 (15)	
F1	0.21626 (18)	−0.02274 (17)	−0.09157 (16)	0.0272 (2)	
F2	0.19296 (18)	0.12317 (17)	0.21936 (15)	0.0282 (2)	
F3	−0.02395 (19)	0.23071 (16)	−0.03724 (18)	0.0278 (2)	
F4	0.4323 (2)	0.5863 (2)	0.38450 (18)	0.0354 (3)	
O1	0.5678 (2)	0.2833 (2)	0.2103 (2)	0.0289 (3)	
O2	0.6023 (3)	0.8243 (2)	0.2410 (2)	0.0266 (3)	
O3	0.7505 (2)	0.5080 (2)	0.0522 (2)	0.0248 (3)	
O4	0.9127 (2)	0.6621 (2)	0.4883 (2)	0.0278 (3)	
O5	0.2346 (3)	0.8900 (2)	0.4430 (2)	0.0296 (3)	
H11	0.636 (6)	0.198 (5)	0.160 (5)	0.055 (3)*	
H12	0.456 (5)	0.233 (5)	0.218 (5)	0.055 (3)*	
H21	0.668 (6)	0.881 (5)	0.196 (5)	0.055 (3)*	
H22	0.653 (6)	0.910 (5)	0.340 (4)	0.055 (3)*	
H31	0.833 (6)	0.586 (5)	0.050 (5)	0.055 (3)*	
H32	0.811 (6)	0.421 (5)	0.027 (5)	0.055 (3)*	
H41	0.885 (6)	0.716 (5)	0.583 (4)	0.055 (3)*	
H42	1.009 (5)	0.644 (5)	0.483 (5)	0.055 (3)*	
H51	0.213 (6)	0.925 (5)	0.365 (4)	0.055 (3)*	
H52	0.344 (6)	0.796 (5)	0.435 (5)	0.055 (3)*	
H6	0.4722	0.5226	0.4629	0.026 (12)*	0.50

### Atomic displacement parameters (Å^2^)

**Table:**

	*U*^11^	*U*^22^	*U*^33^	*U*^12^	*U*^13^	*U*^23^
Cu	0.02240 (14)	0.01519 (13)	0.02078 (14)	0.00360 (8)	0.01130 (9)	0.00657 (9)
Al	0.0176 (3)	0.0141 (3)	0.0194 (3)	0.0027 (2)	0.0093 (2)	0.0063 (2)
F1	0.0279 (5)	0.0262 (5)	0.0377 (6)	0.0082 (4)	0.0226 (5)	0.0129 (5)
F2	0.0273 (5)	0.0301 (6)	0.0225 (5)	−0.0007 (4)	0.0070 (4)	0.0049 (4)
F3	0.0304 (5)	0.0188 (5)	0.0424 (6)	0.0066 (4)	0.0175 (5)	0.0161 (5)
F4	0.0481 (7)	0.0379 (7)	0.0378 (7)	0.0205 (6)	0.0281 (6)	0.0211 (5)
O1	0.0280 (7)	0.0161 (6)	0.0471 (8)	0.0040 (5)	0.0213 (6)	0.0084 (6)
O2	0.0394 (7)	0.0176 (6)	0.0273 (6)	0.0042 (5)	0.0164 (6)	0.0091 (5)
O3	0.0284 (6)	0.0189 (6)	0.0333 (7)	0.0049 (5)	0.0200 (6)	0.0079 (5)
O4	0.0262 (6)	0.0276 (7)	0.0280 (6)	0.0050 (5)	0.0086 (5)	0.0073 (5)
O5	0.0379 (7)	0.0279 (7)	0.0266 (6)	0.0090 (6)	0.0134 (6)	0.0108 (5)

### Geometric parameters (Å, °)

**Table:**

Cu—F4	1.9049 (12)	Al—F1	1.8001 (10)
Cu—O1	1.9441 (14)	Al—F1^ii^	1.8001 (10)
Cu—O2	1.9522 (14)	Al—F2^ii^	1.8091 (11)
Cu—O3	1.9739 (13)	Al—F2	1.8091 (11)
Cu—O4	2.3463 (15)	Al—F3^ii^	1.8209 (11)
Cu—O3^i^	2.7139 (16)	Al—F3	1.8209 (11)
Cu—Cu^i^	3.5440 (4)	F4—F4^iii^	2.596 (3)
			
F4—Cu—O1	86.72 (6)	F4^iii^—F4—O1	75.78 (7)
F4—Cu—O2	90.07 (6)	Cu—F4—O5	120.66 (7)
O1—Cu—O2	172.31 (6)	F4^iii^—F4—O5	123.74 (8)
F4—Cu—O3	172.54 (6)	O1—F4—O5	159.76 (8)
O1—Cu—O3	91.01 (6)	Cu—F4—O2	45.67 (4)
O2—Cu—O3	91.29 (6)	F4^iii^—F4—O2	138.16 (10)
F4—Cu—O4	89.18 (6)	O1—F4—O2	92.70 (6)
O1—Cu—O4	97.22 (6)	O5—F4—O2	75.99 (6)
O2—Cu—O4	89.72 (6)	H11—O1—H12	113 (4)
O3—Cu—O4	98.16 (6)	Cu—O2—Cu^i^	65.57 (4)
F4—Cu—O3^i^	89.91 (5)	F4—O2—Cu^i^	93.18 (5)
O1—Cu—O3^i^	91.35 (6)	Cu—O2—H21	126 (3)
O2—Cu—O3^i^	81.64 (6)	F4—O2—H21	169 (3)
O3—Cu—O3^i^	83.04 (5)	Cu^i^—O2—H21	83 (3)
O4—Cu—O3^i^	171.31 (5)	Cu—O2—H22	117 (3)
F1—Al—F1^ii^	180.00 (8)	F4—O2—H22	91 (3)
F1—Al—F2^ii^	90.44 (5)	Cu^i^—O2—H22	176 (3)
F1^ii^—Al—F2^ii^	89.56 (5)	H21—O2—H22	92 (4)
F1—Al—F2	89.56 (5)	Cu—O3—Cu^i^	96.96 (5)
F1^ii^—Al—F2	90.44 (5)	Cu—O3—H31	123 (3)
F2^ii^—Al—F2	180.0	Cu^i^—O3—H31	107 (3)
F1—Al—F3^ii^	89.97 (5)	Cu—O3—H32	125 (3)
F1^ii^—Al—F3^ii^	90.03 (5)	Cu^i^—O3—H32	106 (3)
F2^ii^—Al—F3^ii^	90.57 (6)	H31—O3—H32	97 (4)
F2—Al—F3^ii^	89.43 (6)	Cu—O4—H41	113 (2)
F1—Al—F3	90.03 (5)	F4—O4—H41	75 (3)
F1^ii^—Al—F3	89.97 (5)	Cu—O4—H42	121 (3)
F2^ii^—Al—F3	89.43 (6)	F4—O4—H42	156 (3)
F2—Al—F3	90.57 (6)	H41—O4—H42	126 (4)
F3^ii^—Al—F3	180.00 (8)	F4—O5—H51	110 (3)
Cu—F4—F4^iii^	109.26 (8)	H51—O5—H52	109 (3)
Cu—F4—O1	47.26 (4)		

Symmetry codes: (i) −*x*+1, −*y*+1, −*z*; (ii) −*x*, −*y*, −*z*; (iii) −*x*+1, −*y*+1, −*z*+1.

### Hydrogen-bond geometry (Å, °)

**Table:**

*D*—H···*A*	*D*—H	H···*A*	*D*···*A*	*D*—H···*A*
O1—H11···F1^iv^	0.87 (3)	1.74 (3)	2.6042 (17)	171 (4)
O1—H12···F2	0.83 (3)	1.86 (3)	2.6899 (18)	176 (4)
O2—H21···F1^i^	0.81 (3)	1.79 (3)	2.6020 (18)	172 (4)
O2—H22···O5^v^	0.83 (3)	1.86 (3)	2.684 (2)	178 (4)
O3—H31···F3^i^	0.77 (3)	1.82 (3)	2.5877 (18)	178 (4)
O3—H32···F3^vi^	0.79 (3)	1.89 (3)	2.6700 (17)	174 (4)
O4—H41···F2^iii^	0.85 (3)	1.95 (3)	2.7840 (19)	169 (4)
O4—H42···O4^vii^	0.67 (3)	2.41 (4)	2.758 (3)	115 (4)
O4—H42···O5^vi^	0.67 (3)	2.41 (4)	2.784 (2)	117 (4)
O5—H51···F2^viii^	0.75 (3)	2.14 (3)	2.8538 (19)	159 (4)
O5—H51···F3^ix^	0.75 (3)	2.50 (3)	3.072 (2)	135 (4)
O5—H52···F4	1.04 (3)	1.67 (3)	2.6620 (19)	158 (3)
F4—H6···F4^iii^	0.9031 (13)	1.6945 (13)	2.596 (3)	175.27 (5)

Symmetry codes: (iv) −*x*+1, −*y*, −*z*; (i) −*x*+1, −*y*+1, −*z*; (v) −*x*+1, −*y*+2, −*z*+1; (vi) *x*+1, *y*, *z*; (iii) −*x*+1, −*y*+1, −*z*+1; (vii) −*x*+2, −*y*+1, −*z*+1; (viii) *x*, *y*+1, *z*; (ix) −*x*, −*y*+1, −*z*.
